# Simplified electrophysiological evaluation of peripheral nerves in critically ill patients: the Italian multi-centre CRIMYNE study

**DOI:** 10.1186/cc5671

**Published:** 2007-01-25

**Authors:** Nicola Latronico, Guido Bertolini, Bruno Guarneri, Marco Botteri, Elena Peli, Serena Andreoletti, Paola Bera, Davide Luciani, Anna Nardella, Elena Vittorielli, Bruno Simini, Andrea Candiani

**Affiliations:** 1 Department of Anesthesiology-Intensive Care, University of Brescia, Spedali Civili, Piazzale Ospedali Civili, 1 – 25123 Brescia, Italy; 2 GiViTI, Gruppo Italiano per la Valutazione degli Interventi in Terapia Intensiva Steering Committee, Aldo e Cele Daccò Clinical Research Centre Mario Negri Institute, Villa Camozzi – 24020 Ranica (BG), Italy; 3 Laboratory of Clinical Epidemiology, Aldo e Cele Daccò Clinical Research Centre Mario Negri Institute, Villa Camozzi – 24020 Ranica (BG), Italy; 4 GiViTI, Gruppo Italiano per la Valutazione degli Interventi in Terapia Intensiva Steering Committee, Villa Camozzi – 24020 Ranica (BG), Italy; 5 Department of Clinical Neurophysiology, University of Brescia, Spedali Civili, Piazzale Ospedali Civili, 1 – 25123 Brescia, Italy

## Abstract

**Introduction:**

Critical illness myopathy and/or neuropathy (CRIMYNE) is frequent in intensive care unit (ICU) patients. Although complete electrophysiological tests of peripheral nerves and muscles are essential to diagnose it, they are time-consuming, precluding extensive use in daily ICU practice. We evaluated whether a simplified electrophysiological investigation of only two nerves could be used as an alternative to complete electrophysiological tests.

**Methods:**

In this prospective, multi-centre study, 92 ICU patients were subjected to unilateral daily measurements of the action potential amplitude of the sural and peroneal nerves (compound muscle action potential [CMAP]). After the first ten days, complete electrophysiological investigations were carried out weekly until ICU discharge or death. At hospital discharge, complete neurological and electrophysiological investigations were performed.

**Results:**

Electrophysiological signs of CRIMYNE occurred in 28 patients (30.4%, 95% confidence interval [CI] 21.9% to 40.4%). A unilateral peroneal CMAP reduction of more than two standard deviations of normal value showed the best combination of sensitivity (100%) and specificity (67%) in diagnosing CRIMYNE. All patients developed the electrophysiological signs of CRIMYNE within 13 days of ICU admission. Median time from ICU admission to CRIMYNE was six days (95% CI five to nine days). In 10 patients, the amplitude of the nerve action potential dropped progressively over a median of 3.0 days, and in 18 patients it dropped abruptly within 24 hours. Multi-organ failure occurred in 21 patients (22.8%, 95% CI 15.4% to 32.4%) and was strongly associated with CRIMYNE (odds ratio 4.58, 95% CI 1.64 to 12.81). Six patients with CRIMYNE died: three in the ICU and three after ICU discharge. Hospital mortality was similar in patients with and without CRIMYNE (21.4% and 17.2%; *p *= 0.771). At ICU discharge, electrophysiological signs of CRIMYNE persisted in 18 (64.3%) of 28 patients. At hospital discharge, diagnoses in the 15 survivors were critical illness myopathy (CIM) in six cases, critical illness polyneuropathy (CIP) in four, combined CIP and CIM in three, and undetermined in two.

**Conclusion:**

A peroneal CMAP reduction below two standard deviations of normal value accurately identifies patients with CRIMYNE. These should have full neurological and neurophysiological evaluations before discharge from the acute hospital.

## Introduction

Critical illness polyneuropathy (CIP) is the commonest and the best-defined neuromuscular alteration seen in the intensive care unit (ICU) [1], affecting 58% of patients with prolonged ICU stay, 70% to 80% of patients with sepsis, septic shock, or multi-organ failure (MOF), and 100% of patients with sepsis and coma [2]. CIP is an axonal polyneuropathy and is a common consequence of systemic inflammatory response syndrome (SIRS) and MOF [3]. In its classic presentation, CIP is a sensory-motor axonal polyneuropathy [1]; however, pure motor and pure sensory forms have also been described [4,5]. CIP is usually suspected in ICU patients who, after a period of days or weeks, cannot be weaned from the ventilator despite the absence of pulmonary or cardiac causes of respiratory failure or because they have various degrees of limb weakness [3]. Neurological signs of CIP may or may not be present at this stage [1]. In addition, neurological examination is often unreliable because of encephalopathy, sedation, or the critical condition of the patient [6]; therefore, comprehensive electrophysiological studies of peripheral nerves are necessary to establish the diagnosis. These should include motor and sensory nerve conduction studies as well as needle electromyography (EMG) in upper and lower limbs [7]. A reduced amplitude of the compound muscle action potential (CMAP) and sensory nerve action potential (SNAP) is the predominant finding; latency and nerve conduction velocity remain normal or are only slightly decreased [7]. Although several studies have prospectively assessed the evolution of CIP [3-5,8-11], they did not start at the time of ICU admission and did not investigate baseline electrophysiological status of peripheral nerves *before *the onset of CIP. Only two small case series have performed electrophysiological investigations in the first ICU days [12,13]. In one study [12], nine patients with SIRS had their initial electrophysiological investigations within a median of five days (range 2 to 25 days) after ICU admission [12]. All showed a CMAP reduction, whereas most SNAPs were normal. In the other study [13], nine patients with moderate to severe multi-organ dysfunction syndrome and SIRS or sepsis had their initial electrophysiological investigations within two to five days after ICU admission. All had a reduction in CMAP (SNAPs were not reported), confirming it as the earliest electrophysiological sign of CIP.

Critical illness myopathy (CIM) is a primary muscle disorder that has been characterised only in recent years [4]. Data on its incidence are lacking, but evidence is mounting that CIM is at least as frequent as CIP [4,14-23]. There is currently substantial consensus about considering CIM as a syndrome with a continuum of myopathic findings [2,24-27]. Differential diagnosis between CIP and CIM is difficult because conventional conduction studies and needle EMG often provide non-specific findings that fail to distinguish between CIM and CIP [28]. Both conditions are characterised by low-amplitude CMAPs and frequently show abnormal spontaneous activity [20,22]. Assessment of recruitment and interference of voluntary EMG pattern is often problematic because of severe weakness or poor voluntary effort in most patients. The differentiating feature may become the SNAP, which may be blunted or masked by the local oedema in critically ill patients, so that these measures are often unreliable [20]. Previous studies have shown that if the patient fails to volitionally activate his/her muscles, electrophysiological diagnosis is invariably CIP even if CIM is ongoing [4,29]. Furthermore, CIM and CIP are frequently associated [4]. We therefore coined the acronym CRIMYNE (critical illness myopathy and/or neuropathy) to define the neuromuscular alterations acquired during the ICU stay. This acronym also identified the current study among the participating centres.

Early diagnosis of CRIMYNE is important for several reasons. Knowing CRIMYNE is present aids managing the ventilator and means the patient has a neuromuscular problem, which is likely to prolong the patient's ventilator dependency and ICU stay [30,31]. In critically ill comatose patients developing tetraparesis or tetraplegia, knowing that CRIMYNE is present may prevent an unreasonably pessimistic prognosis and allows the diagnostician to ascribe paralysis to CRIMYNE rather than to central nervous system deterioration [4]. Early diagnosis combined with serial electrophysiological studies may also be valuable in determining the ultimate prognosis of patients with CRIMYNE and in gauging the rate of recovery, as well as in assessing the effects of treatments such as intensive insulin therapy [32]. However, electrophysiological study is time-consuming, requiring 45 to 90 minutes for its completion [6].

We report a multi-centre, prospective study in a mixed cohort of medical and surgical critically ill adult patients with no evidence of CRIMYNE or MOF at ICU admission who underwent serial clinical and simplified electrophysiological investigations during their entire ICU stay.

The main objective of this study was to evaluate whether a simplified electrophysiological test could accurately diagnose CRIMYNE. Other objectives were to evaluate the onset time of CRIMYNE in relation to ICU admission and to MOF onset, the transition from normal electrophysiology to CRIMYNE, and the evolution of CRIMYNE during the ICU stay.

## Materials and methods

This multi-centre prospective cohort study was performed between January 1998 and March 2001 in nine Italian ICUs belonging to the GiViTI (Gruppo Italiano per la Valutazione degli Interventi in Terapia Intensiva). Local ethics committee approval was obtained beforehand. Written consent was obtained from the patient whenever possible; otherwise, written information was given to their next of kin. Written consent was obtained from all surviving patients as soon as they regained mental competency.

### Inclusion and exclusion criteria

Patients more than 15 years of age whose Simplified Acute Physiology Score II (SAPS II) [33] was between 35 and 70 were eligible for inclusion. This range predicts a risk of developing MOF of more than 30% (unpublished observation by N. Latronico and G. Bertolini derived from intensive care medicine data provided by Rui Moreno, Lisbon, Portugal, and from sepsis study data provided by Martin Langer, Milan, Italy) and a risk of hospital mortality of between 15% and 85% [33].

Exclusion criteria were (a) CRIMYNE or MOF diagnosed within 24 hours of ICU admission, (b) previous neuromuscular disorders, (c) elective surgery, (d) obesity (body mass index of more than 30 kg/m^2^), (e) lower limb disorders precluding nerve conduction study and EMG (for example, oedema, fractures, amputation, plaster casts), and (f) brain death. Centres were allowed to exclude patients if another patient in the same ICU was being concomitantly studied.

### Initial electrophysiological investigations

Twenty-four hours after admission, the SAPS II and Sequential Organ Failure Assessment (SOFA) [34,35] scores were calculated and complete electrophysiological tests performed. These consisted of conventional motor (median and common peroneal nerves) and sensory nerve (median and sural nerves) conduction studies. SNAPs were recorded from the median and sural nerves. For the median nerve, the ring recording electrodes were placed around the proximal (-) and distal (+) interphalangeal joints of the second or third digit; the nerve was stimulated at the wrist, on the volar surface, 2 to 3 cm proximal to the distal crease. For the sural nerve, the surface recording electrodes were placed above (-) and below (+) the lateral malleolus as the nerve passes around it or immediately posteroinferior to the lateral malleolus (-) and 2 to 3 cm distally along the lateral dorsum of the foot (+); the nerve was stimulated along the posterior surface of the leg (calf), slightly lateral to the midline and approximately 10 to 12 cm from the active electrode (-). CMAPs were recorded from the median (abductor pollicis brevis muscle) and common peroneal (extensor digitorum brevis muscle) nerves. For the median nerve, surface recording electrodes were placed over the belly (-) and tendon (+) of the abductor pollicis brevis; the nerve was stimulated at the wrist on the volar surface, 2 to 3 cm proximal to the distal crease and at the elbow over the brachial pulse with the cathode at the volar crease. For the common peroneal nerve, surface recording electrodes were placed over the belly and tendon of the extensor digitorum brevis; the nerve was stimulated over the dorsum of the foot, near the ankle, 7 to 8 cm from the recording electrodes, above (at the lateral popliteal fossa) and below the head of the fibula (below the knee). Incremental electrical stimulation of the nerves was used until the best SNAP or CMAP amplitudes were obtained. If the clinical history and physical examination suggested a median nerve entrapment at the wrist or the median sensory nerve conduction study was abnormal, the median nerve was substituted by the ulnar nerve [36]. The ulnar nerve was stimulated above and below the elbow and the peroneal nerve above and below the head of the fibula to rule out entrapment neuropathies. EMG was recorded using a coaxial needle electrode in the tibialis anterior, quadriceps femori, abductor pollicis brevis, and deltoid muscles; additional muscles were studied in some patients. Impaired neuromuscular transmission due to neuromuscular blocking agents was excluded by 3-Hz stimulation of the distal ulnar nerve. Before electrophysiological tests, heat packs were applied to the skin if its temperature was below 33°C.

A differential diagnosis between CIP, CIM, or combined CIP and CIM was not sought during the ICU stay. Electrophysiological diagnosis of CRIMYNE was achieved if the CMAP or SNAP amplitude of at least two nerves of two limbs was reduced below two standard deviations (SDs) of the lower limit of normality with or without abnormal spontaneous muscle activity [7,12]. Normal values were established in normal control subjects tested in the same laboratory [37] (see Additional file 1). Organ dysfunction was defined according to the SOFA score [34,35]. MOF was defined as the failure of two or more organs in addition to the organ whose failure prompted ICU admission; CIP was not considered as an organ failure for the purpose of defining MOF. SIRS and sepsis were defined according to current standards [38].

### Serial clinical and electrophysiological investigations

**Figure 1:**
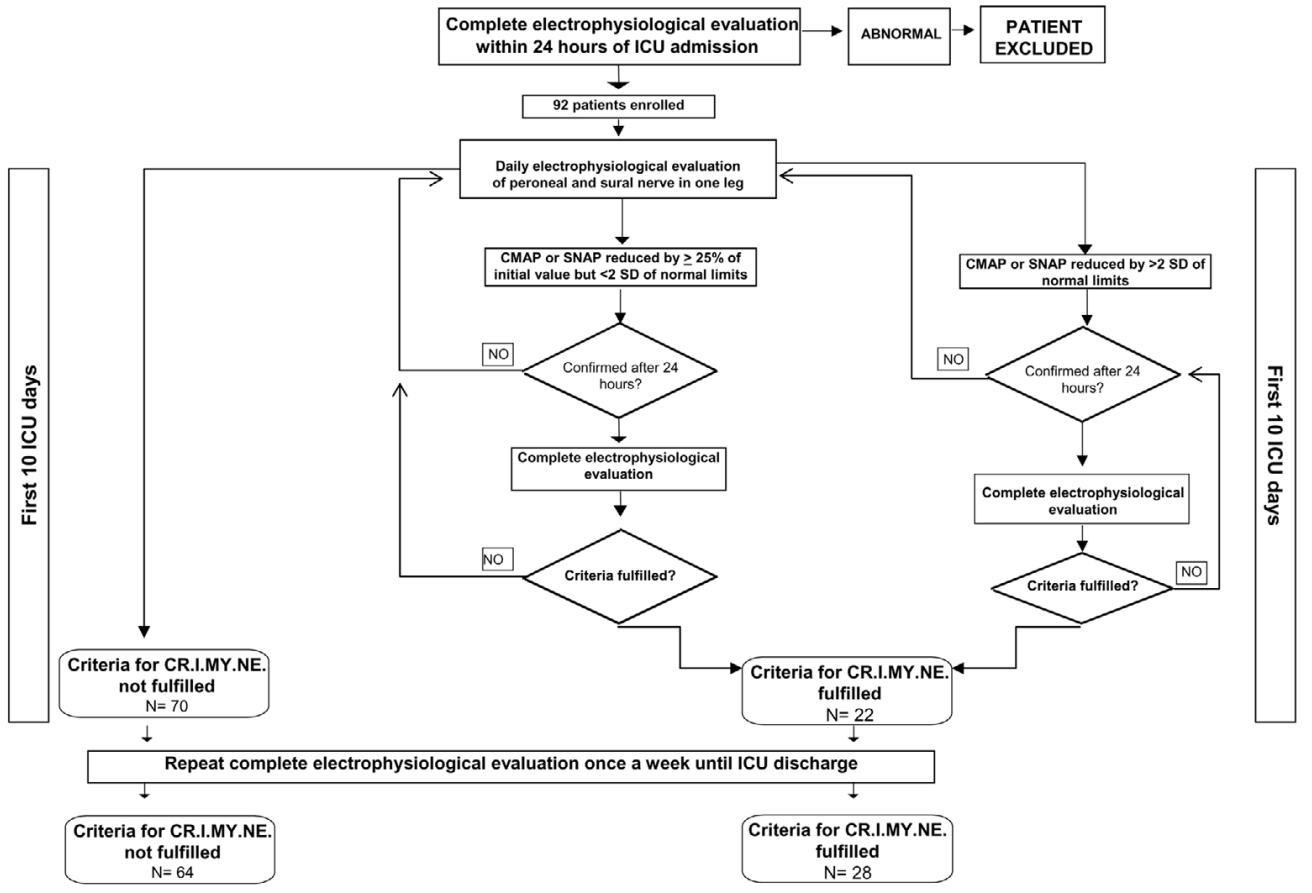
Flow chart of electrophysiological investigations. CMAP, compound muscle action potential; CRIMYNE, critical illness myopathy and/or neuropathy; ICU, intensive care unit; SD, standard deviation; SNAP, sensory nerve action potential.

Daily simplified and weekly complete electrophysiological tests were performed (Figure 1). Simplified electrophysiological tests recorded conduction velocity and amplitude of the sural SNAP and peroneal CMAP in one leg, using surface stimulation and recording electrodes. We arbitrarily defined a 25% decrease from baseline SNAP and CMAP measured at ICU admission as the minimum consistently detectable reduction. If SNAP or CMAP decreased by more than 25% on two consecutive days, a complete electrophysiological test was performed. If the latter was consistent with CRIMYNE, complete weekly electrophysiological tests replaced daily tests until ICU discharge. Otherwise, daily simplified electrophysiological tests were resumed (Figure 1). To minimise artifacts, the same electrode site and size were used for each patient [39].

Patient treatment, including control of blood glucose, conformed to accepted standards. Intravenous insulin (Actrapid HM; Novo Nordisk A/S, Bagsvaerd, Denmark), preferably with the use of a pump, was started if the blood glucose level exceeded 180 mg/dl. The target was a blood glucose level of less than 160 mg/dl. Data on blood glucose level were not collected.

Intensivists and clinical neurophysiologists were unaware of each other's diagnoses. All electrophysiological recordings were re-examined by one author (BG) for quality assessment.

### Follow-up

Patients discharged from the ICU with an electrophysiological diagnosis of CRIMYNE and who were able to cooperate had complete electrophysiological investigations, including sensory and motor nerve conduction studies and EMG of upper and lower limb muscles, before acute hospital discharge. At this stage, a differential diagnosis between CIM, CIP, and combined CIM and CIP was sought.

### Data presentation and statistical analysis

We expressed continuous variables as means (SD) or as medians (interquartile range [IQR]) and discrete variables as counts (percentage) unless otherwise stated. Differences in the study population were analysed by means of a Student's *t *test, Mann-Whitney *U *test, or χ^2 ^test (or Fisher exact test) as appropriate. Ninety-five percent confidence intervals (CIs) were computed for each estimate of interest. The odds ratio (OR) was used to quantify the association between electrophysiological changes and MOF. The times of onset of CIP and MOF, expressed in terms of cumulative incidence, were analysed with Kaplan-Meier curves [40]; comparison was made using the log-rank test. All tests were two-tailed, and a *p *value of less than 0.05 was used to define a statistically significant difference.

## Results

Ninety-two patients were enrolled with a mean monthly enrolment rate of 1.2 patients per ICU. One centre (Brescia, Italy) enrolled 30 patients during the entire study period; the other 8 centres enrolled 4 to 13 patients during 4 to 12 months. Patient characteristics are shown in Table 1.

**Table 1:** Baseline characteristics of the patients

Characteristic	
Total number of patients	92
Age in years	
Median	49.5
Interquartile range	31–67
Absolute range	18–85
Female gender, number (percentage)	29 (31.5)
Simplified Acute Physiology Score II	
Median	42
Interquartile range	38–49
Sequential Organ Failure Assessment score	
Median	7
Interquartile range	6–9
Number of patients artificially ventilated on admission (percentage)	88 (95.7)
Reason for admission, number (percentage)	
Medical	41 (44.6)
Pneumonia	9 (9.8)
Pulmonary oedema	7 (7.6)
Metabolic encephalopathy	6 (6.5)
Post-anoxic encephalopathy	5 (5.4)
Intracranial haemorrhage	5 (5.4)
COPD exacerbation	2 (2.2)
Congestive heart failure	2 (2.2)
Other	5 (5.4)
Emergency surgery	15 (16.3)
Neurosurgery	9 (9.8)
Abdominal surgery	3 (3.3)
Other surgery	3 (3.3)
Trauma	36 (39.1)
Intensive care unit stay in days	
Median	13
Mode (bimodal)	2 (11)
Interquartile range	8–22
Absolute range	1–90

COPD, chronic obstructive pulmonary disease.

The electrophysiological signs of CRIMYNE occurred in 28 patients (30.4%, 95% CI 21.9% to 40.4%) (Table 2), 6 of whom died (3 in the ICU, 3 after ICU discharge). Thirteen of the 92 patients died in the ICU (14.1%) and 4 more died in the hospital after ICU discharge (total of 17 patients [18.5%]). Hospital mortality was similar in patients with and without CRIMYNE (6 patients [21.4%] and 11 patients [17.2%], respectively; Fisher exact test, *p *= 0.771).

**Table 2:** Electrophysiological alterations in the study population

	Time of evaluation		
	
	At diagnosis of CRIMYNE	At ICU discharge	
		Persisting	Resolved
		
Bilateral peroneal CMAP reduction^a^	16 (57%)	13	3
Only bilateral peroneal CMAP	9	7	2
+ unilateral sural SNAP	1	0	1
+ bilateral sural SNAP	2	2	0
+ bilateral sural SNAP + unilateral median CMAP	1	1	0
+ unilateral median SNAP	1	1	0
+ unilateral median CMAP	1	1	0
+ unilateral median SNAP + unilateral median CMAP	1	1	0
Unilateral peroneal CMAP reduction^a^	12 (43%)	5	7
+ unilateral sural SNAP	1	1	0
+ unilateral sural SNAP+ unilateral median SNAP	1	0	1
+ bilateral sural SNAP	2	0	2
+ bilateral sural SNAP + unilateral median CMAP + unilateral median SNAP	2	1	1
+ unilateral median SNAP	3	2	1
+ bilateral median SNAP	1	1	0
+ unilateral median CMAP	2	0	2

^a^Reduction of the CMAP or SNAP amplitude by more than two standard deviations of its normal value. CMAP, compound muscle action potential; CRIMYNE, critical illness myopathy and/or neuropathy; ICU, intensive care unit; SNAP, sensory nerve action potential.

### Time course of CRIMYNE during the ICU stay

An electrophysiological diagnosis of CRIMYNE was preceded by a 25% peroneal CMAP reduction (compared to the baseline value at ICU admission) in all 28 patients (sensitivity 100%); however, the specificity of this abnormality was low (48%) (Table 3). A peroneal CMAP reduction below two SDs of normal values (according to the single centre) had the same sensitivity but better specificity (67%) (Table 3). The more severe the peroneal CMAP reduction, the lower the sensitivity and the higher the specificity (Table 3).

**Table 3:** Sensitivity and specificity of peroneal CMAP reduction to diagnose critical illness myopathy and/or neuropathy

		Time of development	Sensitivity	Specificity
		ICU day	(True-positive rate)	(True-negative rate)
	
	Number (%)	Median (IQR)		
	
1. One peroneal CMAP reduced according to criterion A	64 (69.6)	3 (2–5)	28/28 = 100%	28/64 = 44%
2. One peroneal CMAP reduced according to criterion B	49 (53.3)	4 (2–7)	28/28 = 100%	43/64 = 67%
3. Both peroneal CMAPs reduced according to criterion A	26 (28.3)	6 (3–10)	21/28 = 75%	59/64 = 92%
4. One peroneal CMAP reduced according to criterion A *plus *the contralateral peroneal CMAP reduced according to criterion B	23 (25.0)	6 (3–10)	21/28 = 75%	62/64 = 97%
5. Both peroneal CMAPs reduced according to criterion B	16 (17.4)	6 (3.5–10)	16/28 = 57%	64/64 = 100%

Criterion A = CMAP amplitude reduced by more than 25% of its initial value (at ICU admission) but less than two standard deviations (SDs) of its normal value. Criterion B = CMAP reduced by more than 2 SDs of its normal value. Note that the five categories are not mutually exclusive (for example, the 16 patients in category 5 are also included in category 2). CMAP, compound muscle action potential; ICU, intensive care unit; IQR, interquartile range.

**Figure 2:**
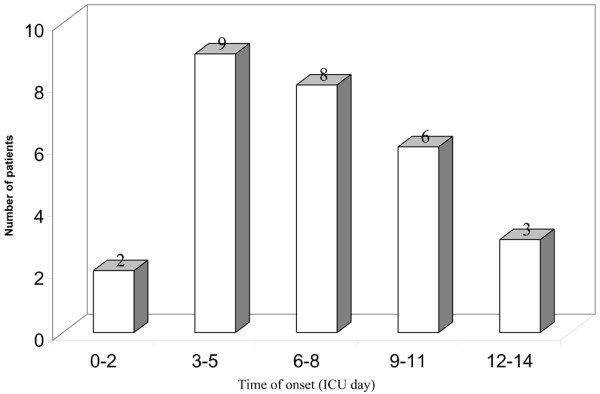
Onset time of critical illness myopathy and/or neuropathy during intensive care unit (ICU) stay.

All 28 patients developed the electrophysiological signs of CRIMYNE within 13 days of ICU admission, 25 (89.3%) within 11 days of ICU admission (Figure 2). The median interval from ICU admission to CRIMYNE was 6 days (95% CI 5 to 9 days, IQR 4 to 10 days).

In 18 patients (64.3%), the amplitude of the nerve action potential amplitude decreased abruptly within 24 hours, and in 10 patients (35.7%) the amplitude dropped progressively over a median of 3.0 days (IQR 2 to 5 days). In 29 patients (31.5%), EMG revealed fibrillation potentials and positive sharp waves, which were evenly distributed among explored muscles. Nerve conduction velocity was normal in all cases. There were no complications specifically attributed to serial electrophysiological measurements.

### Relationship between MOF and CRIMYNE

**Figure 3:**
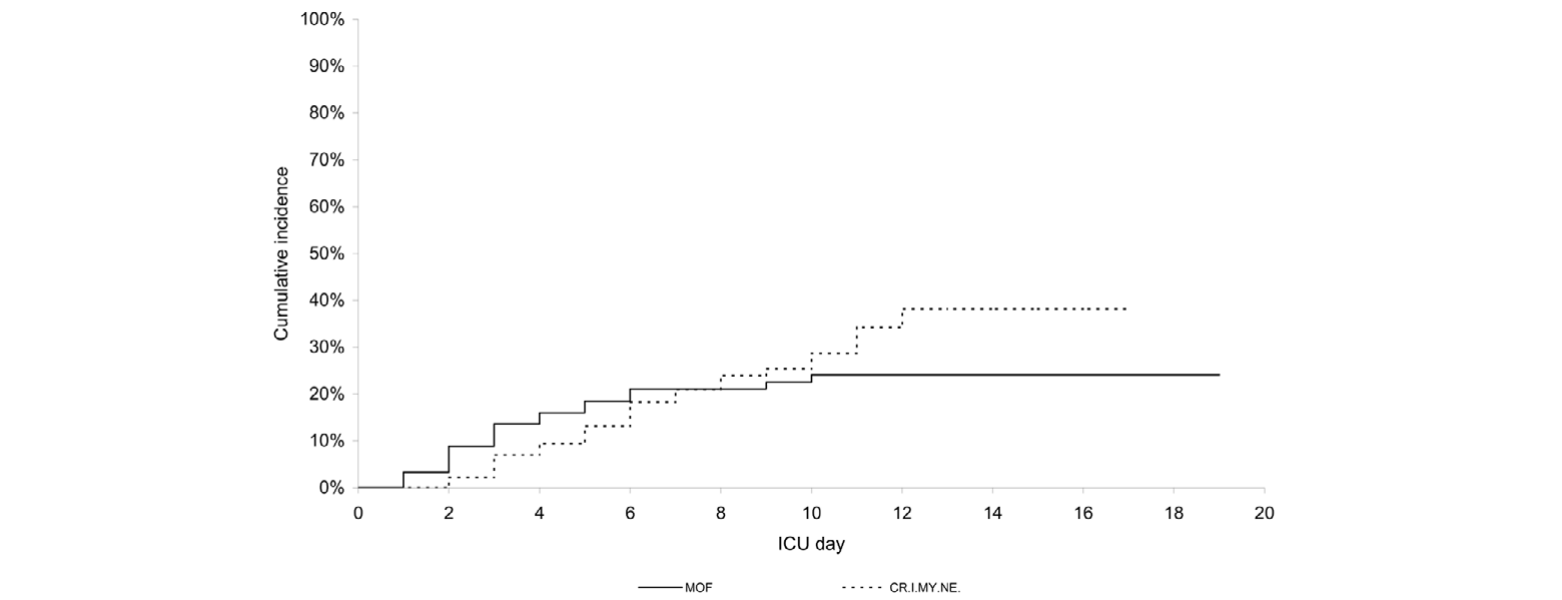
Kaplan-Meier curves comparing the times of onset of critical illness myopathy and/or neuropathy (CRIMYNE) and multi-organ failure (MOF). No difference between the onset times of CRIMYNE and MOF was observed (log-rank test 1.03, *p *= 0.311). ICU, intensive care unit.

MOF occurred in 21 patients (22.8%, 95% CI 15.4% to 32.4%), six of whom died during ICU stay (28.6%). The median interval from ICU admission to MOF was three days (95% CI two to five days, IQR two to five days). Respiratory (17 patients) and cardiovascular (17 patients) failure prevailed and their combination was responsible for the diagnosis of MOF in 12 of the 21 patients (57.1%). There was no difference between the onset times of CRIMYNE and MOF (log-rank test 1.03, *p *= 0.311) (Figure 3).

MOF was strongly associated with CRIMYNE (OR 4.6, 95% CI 1.6 to 12.8): all but two patients with CRIMYNE had single (14 patients) or multiple (12 patients) organ failures. If CRIMYNE were considered an extra organ failure, it would be the most common organ failure in patients with MOF. Furthermore, a diagnosis of MOF would be made in ten (48%) other patients.

### Follow-up

Recovery from CRIMYNE and MOF differed. At ICU discharge, MOF had resolved in all survivors (15 patients), whereas CRIMYNE had resolved in 10 of 28 patients but was still persisting in 18 (64.3%) (Table 2). Of these 18 patients, 3 died after ICU discharge and 2 were unable to volitionally activate their muscles in order to have a complete EMG evaluation. A precise pathological diagnosis was achieved in the 13 remaining patients, which was CIM in six cases, CIP in four, and combined CIM and CIP in three.

## Discussion

CIP and CIM are frequent complications in ICU patients [2] and are responsible for prolonged disability after ICU discharge [41]. Clinical diagnosis is often unreliable in the ICU [1,3,6,7], and therefore electrophysiological studies must be used. Complete electrophysiological investigations are, however, time-consuming [6], and therefore CIP and CIM are rarely systematically investigated in the ICU, except for research purposes. In the present study, we found that a simplified electrophysiological investigation assessment is accurate and can be started early after ICU admission and used in daily routine. The simplified electrophysiological test we used consisted of conduction velocity and amplitude of the sural SNAP and peroneal CMAP in one leg; however, unilateral testing of peroneal CMAP had the best combination of sensitivity and specificity. This is an important finding because the SNAP amplitude is 1,000 times lower than CMAP amplitude and is therefore more difficult to measure accurately, particularly if oedema is present, and is more prone to misinterpretation. Although not formally assessed, the time needed to measure a peroneal CMAP in one leg can be estimated to be 5 to 10 minutes, which is substantially lower than the 45 to 90 minutes needed for a complete electrophysiological investigation [6].

A 25% reduction of the peroneal CMAP was as sensitive as a reduction of more than two SDs in diagnosing CRIMYNE. This first test, however, had a lower specificity (the true-negative rate) and in order to be calculated needed a baseline evaluation of the peroneal CMAP amplitude at ICU admission. The second test proved to be not only more accurate but also more efficient, needing to be compared with *normal *values and not with baseline peroneal CMAP. According to Marciniak and coworkers [37], the possible sources of normal values of electrodiagnostic studies which will permit a report of an abnormal result to be considered reliable include (a) values obtained in a normal group (according to the reference standard) enrolled specifically for the article, (b) normal values established in normal control subjects tested in the same laboratory, and (c) normal values established in normal control subjects using the same electrodiagnostic techniques, even if obtained in another laboratory.

High-sensitivity diagnostic tests have a high negative predictive value and are particularly useful when normal. The test can therefore be proposed as a screening test before a patient's discharge from the ICU or the acute hospital: patients with bilaterally normal peroneal CMAP need no further evaluation; patients with a peroneal CMAP reduction of more than two SDs of normal values, either unilateral or bilateral, are referred to the neurologist for further investigation. The total number of patients to be investigated would vary according to the definition of 'high-risk' critically ill patients – possible definitions are patients with mechanical ventilation longer than three or seven days, patients with sepsis and/or MOF, or patients with a SAPS II of between 35 and 70 [3-5,8-11] – but based on the recruitment rates of this study, it should be in the order of one to two patients per month per ICU.

The fact that primarily the peroneal nerve, a long lower limb motor nerve, was affected has implications for the so-called theory of bioenergetic failure, which is thought to be a relevant pathophysiological mechanism explaining MOF [42] and CIP [3,4,43-45]. In fact, nerve action potential generation and terminal axon structural integrity are critically dependent on axonal transport of proteins and other molecules [46]. Despite their length, axons are devoid of the machinery for biosynthetic processes, and all axonal components are synthesised in the cell body, translocated from the cell body into the axonal process, and then transported to their final destination within the axon [46]. This anterograde transport, particularly the fast transport, requires considerable energy expenditure because material is moved rapidly with rates up to 3 μm/second [46]. If the nerve cell does not receive adequate nourishment due to microcirculatory alterations [47] or the cell cannot use the energy due to cellular dysoxia, the axonal transport fails and distal axonopathy ensues. Bioenergetic failure might explain the extremely rapid decrease of peroneal CMAP observed within 24 hours of normal CMAP in 18 (64.3%) of our patients, which represents a substantial divergence from the traditional observation that at least one week is needed for axonal neuropathy to become apparent. Although these CMAP changes could be due to a combination of dysfunction of both peripheral nerves and muscles, the important message is that functional derangement happened very early, confirming a hypothesis we proposed 11 years ago [4]. This early functional derangement may be an important biological sign in critically ill patients and, as Bolton noted [48], could be used in research aiming at interrupting pathological mechanisms at their onset.

We did not find an association between CIP and SIRS, sepsis, drugs, or nutrition. Because blood glucose data were not collected, association with hyperglycaemia could not be confirmed. Conversely, the risk of having CIP was almost five times greater in patients with MOF than in patients without, a result in agreement with a recent systematic review [49] and a prospective multi-centre cohort study [21]. Several previous studies reported an association between CIP and sepsis or MOF, although they selectively included patients with sepsis [4,9,10,50] or with sepsis and MOF [5], used non-validated MOF-scoring systems [3,5,8,9], or did not provide details of criteria used to diagnose MOF [3,11,13]. Zochodne and colleagues [3] first observed that CIP developed during the course of MOF and improved in some patients as the critical illness subsided, and they suggested that the pathogenesis of failing systemic organs and peripheral nerve damage might be the same. Indeed, the strong association between CIP and MOF and the similarity of their onset times suggest that CIP itself could be considered an organ failure: that of the peripheral nervous system.

In our study, hospital mortality was not different in patients with and without CIP, a result in contrast with two previous studies [9,11]. In the study by Leijten and colleagues [9] of critically ill patients mechanically ventilated more than seven days, the hospital mortality was more than double in patients with CIP (48%) than in patients without (19%; *p *= 0.03); however, mortality was no longer significantly different at 1 year (52% and 43% in patients with and without CIP, respectively; *p *= 0.18). Garnacho-Montero and colleagues [11] studied a very select population of patients with sepsis, MOF, and a duration of mechanical ventilation of more than nine days. A significant proportion of patients had extremely severe derangement of physiological variables and 40% had septic shock [41]. Hospital mortality was higher in patients with CIP than in patients without (84% versus 56.5%, respectively; *p *= 0.01). These figures are much higher than ours and suggest that differences in patients' case mix may have accounted for the difference. However, we cannot exclude the possibility that the small number of events in our study population precluded a thorough statistical evaluation.

The simplified electrophysiological test used in our study could not and cannot distinguish CIM from CIP [20,22-24,28]. We were able to achieve a precise pathological diagnosis in only 13 of 28 (46%) patients after ICU discharge. Nine (69%) of them were found to have CIM alone or in combination with CIP, confirming that CIM is an often-overlooked diagnosis. We cannot exclude the fact that a higher number of patients would have been diagnosed with CIM if we had used muscle biopsy [4], myosin/actin ratio [51], or specialised electrophysiological investigations such as direct muscle stimulation [20,22-24]. Recently, a diagnostic algorithm for differentiating CIM from CIP which combines direct muscle stimulation and conventional techniques was proposed [23]; however, differential diagnosis between CIP and CIM during ICU stay is of unproven relevance.

### Potential pitfalls of the simplified electrophysiological test

Acute peroneal palsy, tissue oedema, and advanced age (particularly more than 70 years) may cause true or artifactual peroneal CMAP reduction. Acute peroneal nerve palsy is most commonly caused by trauma, surgery, or compression of the nerve trunk at the fibular head [52]. Isolated non-traumatic lesions are rare. In many patients, however, the cause remains undetermined and in the absence of other signs is often assumed to be due to transient compression. Motor conduction across the segment of fibula head is particularly important in distinguishing patients with peroneal neuropathy at this level from patients with other lower-extremity neurological disorders (class III and class IV evidence) [37]. Inadequate consideration of these potential pitfalls may substantially increase the number of false-positive cases of CRIMYNE; however, acute peroneal entrapment neuropathies are a cause of disability which deserves medical attention.

## Conclusion

Assessment of the peroneal nerve CMAP amplitude before discharge from the ICU is feasible and can be implemented in clinical routine. A peroneal CMAP reduction of more than two SDs of normal value accurately identifies patients with CRIMYNE. These patients should have full neurological and neurophysiological evaluations before discharge from the acute hospital. Future availability of low-cost simplified EMG machines would be desirable for promoting the widespread use of this important non-invasive diagnostic test in the ICU.

## Key messages

• A peroneal CMAP reduction of more than two SDs of normal value accurately identifies patients with CRIMYNE.

• Transition from normal peripheral nerve electrophysiology to CRIMYNE can be extremely rapid (24 hours).

• CRIMYNE, once diagnosed, persists in the majority of patients at ICU discharge.

• CRIMYNE is associated with MOF, not with SIRS or sepsis.

• CRIMYNE is not associated with increased hospital mortality.

## Abbreviations

CI = confidence interval; CIM = critical illness myopathy; CIP = critical illness polyneuropathy; CMAP = compound muscle action potential; CRIMYNE = critical illness myopathy and/or neuropathy; EMG = electromyography; ICU = intensive care unit; IQR = interquartile range; MOF = multi-organ failure; OR = odds ratio; SAPS II = simplified acute physiology score II; SD = standard deviation; SIRS = systemic inflammatory response syndrome; SOFA = sequential organ failure assessment; SNAP = sensory nerve action potential.

## Competing interests

NL, GB, and BS are part of the Steering Committee of the GiViTI (Gruppo Italiano per la Valutazione degli Interventi in Terapia Intensiva), which is the recipient of an unconditional grant from AstraZeneca Italia S.p.A. (Basiglio, Italy), Sanofi-Aventis (Paris, France), and Draeger Italia (Corsico, Italy). The other authors declare that they have no competing interests.

## Authors' contributions

All authors made a substantial contribution to the study design and methods. NL conceived the idea of the study. NL, GB, and BG designed the protocol. GB and DL performed the statistical analyses. BG was responsible for neurophysiological investigations of the study. NL, MB, EP, SA, PB, AN, and EV were responsible for the clinical investigations of the study. NL drafted the manuscript and all other authors critically revised it for important intellectual content. All authors read and approved the final manuscript.

## Supplementary Material

Additional file 1 A table showing the normal mean value and lower limit of normality of motor and sensory nerve conduction studies in the nine participating centres. Click here for file
