# Supplementary material for: Simplified electrophysiological evaluation of peripheral nerves in critically ill patients: the Italian multi-centre CRIMYNE study
Source: Crit Care. 2007 Jan 25;11(1):R11. doi: 10.1186/cc5671 (PMC2151880; doi:10.1186/cc5671)
Supplement: Additional file 1 — A table showing the normal mean value and lower limit of normality of motor and sensory nerve conduction studies in the nine participating centres. [file cc5671-S1.doc]

**Additional Table. Normal mean value and lower limit of normality of motor and sensory nerve conduction studies in the 9 participating centres.**

Lower limit of normality (-2SD) is two standard deviations below the normal mean value. MCV= motor conduction velocity; CMAP= compound muscle action potential; SCV= sensory conduction velocity; SNAP= sensory nerve action potential.

| Centre No. |  | Peroneal nerve | | Median motor nerve | |  | Sural nerve | | Median sensory nerve | |
| --- | --- | --- | --- | --- | --- | --- | --- | --- | --- | --- |
|  |  | MCV  (m/sec) | CMAP amplitude  (mV) | MCV  (m/sec) | CMAP amplitude  (mV) |  | SCV  (m/sec) | SNAP amplitude  (micronV) | SCV  (m/sec) | SNAP amplitude  (micronV) |
| 1 | Mean | 51.64 | 13.54 | 57.18 | 15.66 |  | 47.74 | 18.82 | 55.20 | 14.78 |
|  | SD | 4.28 | 4.14 | 4.47 | 4.65 |  | 3.42 | 5.86 | 4.68 | 4.64 |
|  | -2SD | 43.08 | 5.26 | 48.24 | 6.36 |  | 40.90 | 7.10 | 45.84 | 5.50 |
| 2 | Mean | 50.90 | 8.10 | 62.70 | 14.00 |  | 45.00 | 18.90 | 56.20 | 18.50 |
|  | SD | 4.70 | 2.30 | 7.00 | 4.60 |  | 2.50 | 6.70 | 5.80 | 5.60 |
|  | -2SD | 41.50 | 3.50 | 48.70 | 4.80 |  | 40.00 | 5.50 | 44.60 | 7.30 |
| 3 | Mean | 50.42 | 12.90 | 55.70 | 12.90 |  | 46.40 | 16.40 | 56.91 | 19.60 |
|  | SD | 4.12 | 4.02 | 3.90 | 4.42 |  | 3.70 | 5.40 | 4.00 | 5.50 |
|  | -2SD | 42.18 | 4.86 | 47.90 | 4.06 |  | 39.00 | 5.60 | 48.91 | 8.60 |
| 4 | Mean | 49.10 | 9.10 | 57.40 | 10.30 |  | 50.10 | 15.44 | 56.00 | 18.00 |
|  | SD | 3.97 | 2.80 | 3.47 | 3.40 |  | 4.00 | 5.15 | 4.70 | 4.80 |
|  | -2SD | 41.16 | 3.50 | 50.46 | 3.50 |  | 42.10 | 5.14 | 46.60 | 8.40 |
| 5 | Mean | 48.80 | 7.00 | 57.50 | 9.00 |  | 51.00 | 17.50 | 56.00 | 15.30 |
|  | SD | 3.70 | 1.80 | 4.90 | 2.70 |  | 5.80 | 5.80 | 5.80 | 4.80 |
|  | -2SD | 41.40 | 3.40 | 47.70 | 3.60 |  | 39.40 | 5.90 | 44.40 | 5.70 |
| 6 | Mean | 49.50 | 8.90 | 56.70 | 13.20 |  | 48.30 | 17.20 | 52.80 | 20.50 |
|  | SD | 3.80 | 2.50 | 4.90 | 4.30 |  | 4.00 | 5.50 | 4.80 | 6.80 |
|  | -2SD | 41.90 | 3.90 | 46.90 | 4.60 |  | 40.30 | 6.20 | 43.20 | 6.90 |
| 7 | Mean | 51.90 | 11.30 | 58.00 | 21.00 |  | 51.00 | 15.50 | 61.10 | 21.80 |
|  | SD | 4.50 | 3.30 | 4.20 | 7.00 |  | 4.50 | 5.50 | 5.50 | 6.40 |
|  | -2SD | 42.90 | 4.70 | 49.60 | 7.00 |  | 42.00 | 4.50 | 50.10 | 9.00 |
| 8 | Mean | 51.40 | 8.80 | 55.90 | 9.70 |  | 48.70 | 14.80 | 59.40 | 19.00 |
|  | SD | 4.10 | 2.50 | 2.60 | 3.00 |  | 3.60 | 4.80 | 4.90 | 6.20 |
|  | -2SD | 43.20 | 3.80 | 50.70 | 3.70 |  | 41.50 | 5.20 | 49.60 | 6.60 |
| 9 | Mean | 48.50 | 8.90 | 58.30 | 12.30 |  | 46.40 | 16.40 | 56.30 | 20.90 |
|  | SD | 2.80 | 3.00 | 5.20 | 4.20 |  | 2.40 | 5.50 | 4.00 | 7.10 |
|  | -2SD | 42.90 | 2.90 | 47.90 | 3.90 |  | 41.60 | 5.40 | 48.30 | 6.70 |
